# Whole-Spine MRI Reveals High Prevalence of Multifocal Spondylodiscitis and Identifies a High-Risk Subgroup: A Retrospective Cohort Study of 274 Patients

**DOI:** 10.3390/medicina62050989

**Published:** 2026-05-19

**Authors:** Steffen Heinrich Schulz, Franz-Joseph Dally, Johannes Vogel, Peter Fennema, Moritz Kolster, Frederic Bludau

**Affiliations:** 1 Orthopedic and Trauma Surgery Center, University Medical Center Mannheim, University of Heidelberg, Theodor-Kutzer-Ufer 1-3, 68167 Mannheim, Germany; franz.dally@umm.de (F.-J.D.); johannes.vogel@umm.de (J.V.); moritz.kolster@umm.de (M.K.); frederic.bludau@umm.de (F.B.); 2 AMR Advanced Medical Research, 8708 Maennedorf, Switzerland; p.fennema@amr-cro.com

**Keywords:** spondylodiscitis, multifocal, whole-spine MRI, mortality, *Staphylococcus aureus*, spinal infection

## Abstract

*Background and Objectives*: Spondylodiscitis is a severe spinal infection associated with substantial mortality. Standard diagnostic imaging is often limited to the symptomatic spinal segment, which may fail to detect infection foci in other spinal regions. The prevalence and prognostic significance of multifocal spondylodiscitis remain insufficiently characterized. *Materials and Methods*: A retrospective single-center cohort study was conducted at the University Medical Center Mannheim, Germany. All patients with a first diagnosis of imaging-confirmed infectious spondylodiscitis treated between 2008 and 2017 were included (n = 274). Disease distribution was classified as monosegmental, multisegmental unifocal, or multifocal. The study evaluated the detection rate of multifocal disease stratified by imaging modality (whole-spine MRI vs. segmental MRI) and assessed in-hospital mortality according to disease distribution, comorbidity burden, and pathogen type. *Results*: Among the 139 patients who underwent whole-spine MRI, multifocal spondylodiscitis was identified in 25 (18.0%) compared with 2 out of 116 patients (1.7%) who received segmental MRI. Overall in-hospital mortality was 16.9% (46/272). Mortality was substantially higher in patients with multifocal disease (40.0%) than in those with monosegmental (13.7%) or multisegmental unifocal involvement (15.6%, *p* = 0.002). Increasing comorbidity burden (7.5% with no comorbidities to 27.1% with three or more; *p* = 0.008) and *Staphylococcus aureus* infection (26.2% vs. 11.0%; *p* = 0.010) were also significantly associated with mortality. *Conclusions*: Multifocal spondylodiscitis was more frequently detected with whole-spine MRI and was associated with substantially increased in-hospital mortality. These findings support consideration of a low threshold for whole-spine MRI in the primary diagnostic workup of suspected spondylodiscitis. Further prospective studies are required to confirm these findings.

## 1. Introduction

Spondylodiscitis is a severe infection of the intervertebral disc and adjacent vertebral bodies and is associated with considerable morbidity and substantial mortality reported between 5% and 25% in the literature [1,2,3,4]. Despite advances in diagnostic imaging, antimicrobial therapy, and surgical management, the disease remains clinically challenging [5]. The global incidence is rising, driven by population aging, increasing prevalence of predisposing comorbidities, and improved diagnostic detection [1,2,6,7]. Delayed diagnosis may adversely affect outcome [5,6].

Magnetic resonance imaging (MRI) is considered the imaging modality of choice in the diagnostic workup of suspected infectious spondylodiscitis, with a reported sensitivity of approximately 96% and specificity of 93% [4,8,9,10]. MRI allows for the early detection of vertebral and discal infection and facilitates the assessment of associated epidural or paravertebral abscess formation [4,8,9]; alternative modalities such as CT or bone scintigraphy are used when MRI is contraindicated [7].

In routine clinical practice, however, diagnostic imaging is often limited to the spinal region suggested by the predominant symptoms or preliminary findings. This segment-oriented approach may fail to identify infectious lesions in other spinal regions. Multifocal spondylodiscitis, in which two or more different spinal regions are involved, has been reported in the literature with prevalence estimates ranging from approximately 4% to 13% [1,11,12]. However, the frequency and prognostic significance of multifocal involvement remain insufficiently characterized.

For spine surgeons, this question is clinically important because the underestimation of disease extent may affect both risk stratification and treatment planning. Knowledge of the precise localization and extent of destruction directly influences therapeutic decision-making in spinal infection, including the need for further diagnostic workup, multidisciplinary management, and the scope of surgical intervention in selected cases [13,14,15].

The aim of the present retrospective single-center study was therefore to evaluate the distribution of infectious spondylodiscitis within the spine, with particular focus on multifocal involvement, and to assess whether whole-spine MRI was associated with the increased detection of multifocal disease compared with segmental MRI. In addition, we examined the microbiological spectrum, the relationship between comorbidity burden and outcome, and whether the pattern of spinal involvement was associated with in-hospital mortality in a cohort of 274 patients treated at a university center over a 10-year period.

## 2. Methods

### 2.1. Study Design and Setting

This retrospective single-center cohort study was conducted at the Orthopedic and Trauma Surgery Center of the University Medical Center Mannheim, Germany, a university hospital providing tertiary care. The retrospective patient cohort covered the period from 2008 to 2017. The ethics application was submitted on 4 February 2019. Ethics approval was granted on 12 February 2019. Data extraction and scientific analysis were performed after ethics approval in 2019. No prospective patient recruitment or intervention was performed, as this was a retrospective observational study based on existing clinical records and imaging data.

### 2.2. Patient Identification and Eligibility

Patients were identified from institutional clinical documentation (SAP hospital information system) and the radiology database (SyngoVia; Siemens Medical, Erlangen, Germany). All potentially eligible cases were subsequently reviewed by chart and imaging assessment. Inclusion criteria were: (1) diagnosis of infectious spondylodiscitis confirmed by imaging, (2) first diagnosis of spondylodiscitis, and (3) patients of any age and either sex. Patients with chronic spondylodiscitis were excluded because the spatial pattern of disease involvement at first presentation could not be assessed reliably in longstanding disease. After application of the eligibility criteria, 274 patients were included in the study cohort.

### 2.3. Imaging Assessment

Imaging findings at the time of primary diagnostic workup were reviewed. Since 2015, the institution’s orthopedic trauma center has implemented a standardized whole-spine MRI protocol for patients with clinical and laboratory suspicion of spondylodiscitis [10]. This protocol includes at least a sagittal whole-spine short tau inversion recovery (STIR) sequence as a screening tool for multifocal involvement. However, because this was an interdisciplinary real-world cohort, some patients underwent only segmental MRI of the cervical, thoracic, or lumbar spine, and patients with contraindications to MRI (e.g., non-MRI-conditional cardiac devices) underwent alternative imaging such as CT or bone scintigraphy. For analysis, imaging modality was categorized as whole-spine MRI, segmental MRI, or other imaging (including CT and bone scintigraphy).

### 2.4. Variables and Definitions

The following variables were extracted from the medical records: sex; age category (≤50 years, 51–70 years, >70 years); comorbidities and risk factors, including diabetes mellitus, immunosuppressive therapy, malignant disease, renal disease, cardiovascular disease, liver disease, and substance abuse; previous spinal procedures or interventions; imaging modality and coverage; spinal localization of infection; microbiological findings from blood cultures, CT-guided biopsy, or intraoperative specimens; and in-hospital mortality.

Disease distribution was classified into three categories: monosegmental (single motion segment involved), multisegmental unifocal (multiple contiguous segments within the same spinal region), and multifocal (infectious involvement in two or more different spinal regions).

### 2.5. Outcomes

The study evaluated the detection rate of multifocal spondylodiscitis stratified by imaging modality (whole-spine MRI vs. segmental MRI vs. other imaging) and in-hospital mortality according to disease distribution, with particular focus on multifocal disease. Additional descriptive analyses included localization, microbiological spectrum, comorbidity burden, pathogen-specific mortality, and previous spinal surgery or intervention.

### 2.6. Statistical Analysis

Data were analyzed descriptively using frequency distributions and percentages. Associations between categorical variables were assessed using the chi-square test. Where applicable, Cramér’s V was used as a measure of effect size. A two-sided *p*-value of <0.05 was considered statistically significant. Statistical analyses were performed using IBM SPSS Statistics version 22.0.0.0 (IBM Corp., Armonk, NY, USA) and Microsoft Excel.

## 3. Results

### 3.1. Patient Characteristics and Comorbidity Burden

A total of 274 patients with imaging-confirmed infectious spondylodiscitis were included. Of these, 166 patients (60.6%) were male and 108 (39.4%) were female. The cohort was elderly: 193 patients (70.4%) were older than 70 years, 59 (21.5%) were aged 51–70 years, and 22 (8.0%) were 50 years or younger (Table 1).

Multimorbidity was highly prevalent. Among the 268 patients with complete comorbidity data, 228 (85.1%) had at least one documented comorbidity, and 70 (26.1%) had three or more. The most common conditions were cardiovascular diseases (155 patients, 56.6%), diabetes mellitus (75, 27.4%), malignant disease (72, 26.9%), renal disease (58, 21.6%), immunosuppressive conditions (56, 20.4%), and liver disease or substance abuse (35, 12.8%). A total of 65 patients (23.7%) had undergone prior spinal surgery or spinal injection in the affected region before the index diagnosis.

### 3.2. Imaging Modalities

At the time of primary diagnostic workup, 255 out of 274 patients (93.0%) underwent MRI, whereas 19 patients (6.9%) were assessed using other imaging modalities, mainly CT or bone scintigraphy, typically because MRI was contraindicated. Among the 255 patients who underwent MRI, 139 (50.7% of the total cohort) received whole-spine MRI and 116 (42.3%) underwent segmental MRI of the cervical, thoracic, or lumbar spine.

### 3.3. Pathogen Spectrum

Among the 270 patients with available microbiological data, a causative pathogen was isolated in 161 (59.6%), whereas no organism was identified in 109 cases (40.4%), most likely attributable to prior antibiotic treatment or insufficient biopsy yield. *Staphylococcus aureus* was the most frequently isolated organism, found in 65 out of 161 culture-positive patients (40.3%), followed by *Staphylococcus epidermidis* in 19 (11.8%), *Escherichia coli* in 13 (8.1%), and methicillin-resistant *S. aureus* (MRSA) in 9 (5.6% of culture-positive cases). Tuberculous spondylodiscitis was diagnosed in 3 cases and fungal infection in an additional 3 cases (each 1.9% of culture-positive cases). Other organisms, including enterococci, streptococci, and Gram-negative rods, accounted for the remaining isolates.

### 3.4. Distribution of Disease and Detection of Multifocal Involvement

Overall, infection was classified as monosegmental in 198 patients (72.3%), multisegmental unifocal in 46 patients (16.8%), and multifocal in 30 patients (10.9%). Among the patients with monosegmental disease, the lumbar spine was the most commonly affected region, accounting for 103 out of 198 cases (52.0%). Thoracic involvement was present in 39 cases (19.7%), cervical involvement in 15 cases (7.6%), and transitional regions were affected less frequently, including the cervicothoracic junction in 4, the thoracolumbar junction in 14, and the lumbosacral junction in 23 cases.

The key imaging finding was the substantial difference in the detection of multifocal spondylodiscitis according to MRI strategy. Among the patients who underwent whole-spine MRI, 25 out of 139 (18.0%) were found to have multifocal disease. In contrast, only 2 out of 116 patients (1.7%) who underwent segmental MRI were identified as having multifocal involvement. An additional 3 multifocal cases were detected by other imaging modalities (CT or bone scintigraphy), yielding 30 multifocal cases in total.

When disease distribution was analyzed by imaging modality, whole-spine MRI identified monosegmental disease in 84 patients (60.4%), multisegmental unifocal disease in 30 (21.6%), and multifocal disease in 25 (18.0%). In the segmental MRI group, the corresponding proportions were 98 (84.5%), 16 (13.8%), and 2 (1.7%). In the group assessed by other imaging modalities, 16 out of 19 patients (84.2%) had monosegmental disease and 3 (15.8%) had multifocal disease, whereas none were classified as multisegmental unifocal (Table 2).

### 3.5. In-Hospital Mortality

Overall in-hospital mortality during the index admission was 16.9% (46 of 272 patients with available outcome data). Male mortality was 17.6% and female mortality 15.9%; this difference was not statistically significant (*p* = 0.771) (Table 3).

The pattern of spinal involvement had a significant association with mortality (*p* = 0.002, Cramér’s V = 0.22). Patients with multifocal disease had a mortality rate of 40.0% (12/30) compared with 13.7% (27/197) in monosegmental disease and 15.6% (7/45) in multisegmental unifocal disease.

Comorbidity burden was also significantly associated with mortality (*p* = 0.008, Cramér’s V = 0.20). Mortality rose progressively from 7.5% in patients with no comorbidities (3/40) to 10.1% in those with one (10/99), 20.3% with two (12/59), and 27.1% in patients with three or more comorbidities (19/70).

**Figure 1:**
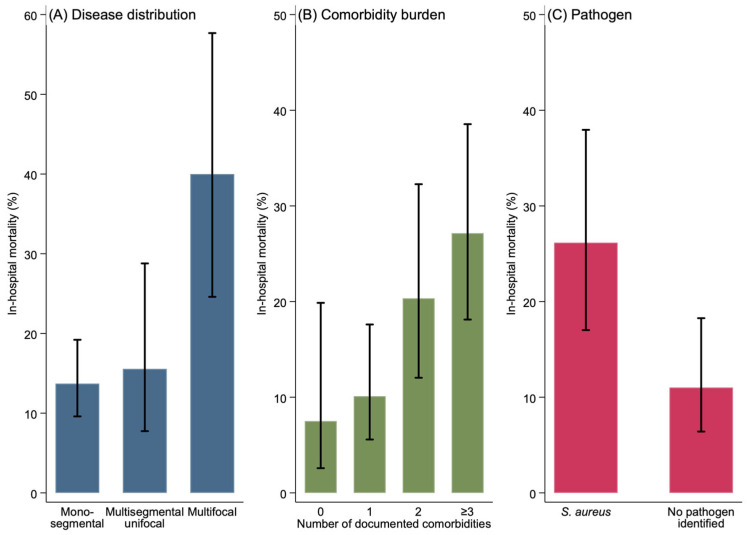
In-hospital mortality stratified by (**A**) disease distribution (monosegmental, multisegmental unifocal, multifocal); (**B**) comorbidity burden (0, 1, 2, ≥3 documented comorbidities); and (**C**) pathogen (*Staphylococcus aureus* vs. no pathogen identified). Bars represent mortality (%); whiskers represent Wilson 95% confidence intervals. *p*-values from chi-square testing: (**A**) *p* = 0.002; (**B**) *p* = 0.008; (**C**) *p* = 0.010. Exact numerical values, denominators, and effect-size estimates are provided in Table 3.

Among pathogen-specific analyses, *S. aureus* infection was associated with significantly higher mortality. Of the 65 patients with *S. aureus* infection, 17 died (26.2%) compared with 12 out of 109 patients (11.0%) in whom no pathogen was identified (*p* = 0.010, Cramér’s V = 0.20). These mortality patterns are summarized in Figure 1.

## 4. Discussion

The most important finding of the present study is that whole-spine MRI was associated with a substantially higher detection rate of multifocal spondylodiscitis than segmental MRI. In our cohort, multifocal disease was identified in 25 out of 139 patients who underwent whole-spine MRI (18.0%) compared with only 2 out of 116 patients who underwent segmental MRI (1.7%); overall, 30 out of 274 patients (10.9%) had multifocal disease. This finding directly addresses the diagnostic gap regarding whether infectious lesions in other spinal regions may remain unrecognized when imaging is restricted to the clinically suspected spinal region.

From a spine-surgical perspective, this observation is clinically relevant because multifocal disease was associated with substantially increased in-hospital mortality. Whereas overall mortality was 16.9%, and mortality in monosegmental and multisegmental unifocal disease was 13.7% and 15.6% respectively, patients with multifocal involvement had a mortality rate of 40.0% (*p* = 0.002).

This interpretation is consistent with the broader literature, which describes spondylodiscitis as a condition associated with substantial mortality, particularly in multimorbid and systemically compromised patients [3,6,16]. Notably, our cohort appears older and more multimorbid than many previously published series, which generally describe spondylodiscitis as occurring predominantly in patients older than 50 years or in the fifth to seventh decades of life [1,2]. In our population, 70.4% of patients were older than 70 years, and 85.1% had at least one documented comorbidity. This older and more multimorbid patient profile likely reflects broader epidemiological trends, including population aging and the increasing number of patients living with chronic diseases. It may also partially explain the relatively high overall mortality rate of 16.9% observed in our cohort compared with some prior reports. Our findings extend the concept of vulnerability by framing multifocal spinal involvement as a prognostic marker that identifies a vulnerable subgroup. Multifocal disease most likely reflects more advanced or disseminated infection at presentation. It marks a high-risk patient rather than a target for direct intervention.

The progressive increase in mortality from 7.5% in patients without comorbidities to 27.1% in those with three or more comorbidities is consistent with our data and with the broader spondylodiscitis literature indicating worse outcomes in multimorbid patients [3,6]. Similarly, the significantly higher mortality associated with *S. aureus* infection (26.2% vs. 11.0%, *p* = 0.010) is in agreement with reports identifying *S. aureus* as a predominant pathogen in pyogenic spondylodiscitis and linking it to worse outcome or treatment failure [6,8]. Together, these findings indicate that multifocal involvement, higher comorbidity burden, and S. aureus infection were each associated with increased in-hospital mortality.

The comparison with the recent literature further supports the relevance of our findings. Henkelmann et al. reported multifocal involvement in approximately 13% of 79 patients treated at a German university center, with an overall mortality of 10% [11]. Notably, that study did not find a significant association between multifocal disease and mortality, which may reflect its smaller sample size and lower overall event rate. In contrast, our cohort of 274 patients showed a statistically significant association between multifocal disease and higher in-hospital mortality.

The practical implication is that missed multifocal disease may have consequences beyond incomplete radiological assessment. Underestimation of disease extent may influence the intensity of diagnostic workup, the perceived severity of infection, inpatient monitoring, the search for disseminated or synchronous infectious foci, and, in selected cases, the scope of surgical planning. Undetected secondary foci may lead to the underestimation of disease extent and thereby complicate treatment planning. Even in patients managed conservatively, accurate knowledge of the spatial distribution of infection is relevant for clinical decision-making.

Our results also support a more nuanced discussion of the optimal imaging strategy in suspected spondylodiscitis. Current diagnostic guidance does not clearly define whether multifocal disease should be systematically excluded during the initial workup. In this context, our data lend support to considering a low threshold for whole-spine MRI, particularly when clinical presentation is severe, symptoms are poorly localizing, or the identification of multifocal disease would meaningfully alter management. Given the retrospective and non-randomized design, however, these observations provide initial evidence rather than definitive grounds for changing routine practice. Our experience with a standardized whole-spine MRI protocol introduced in 2015 suggests that such an approach can be integrated into clinical practice at a university hospital.

From a feasibility perspective, in our institutional workflow, the addition of a sagittal whole-spine STIR sequence to the standard MRI protocol required only limited additional scanner time, and was therefore practicable in routine clinical use at our tertiary center. The cost-effectiveness of a routine whole-spine approach in suspected spondylodiscitis has, however, not been formally evaluated. In settings with more limited MRI capacity, prospective evaluation should weigh the additional scanner time against the yield of detecting multifocal disease and its downstream clinical impact.

At the same time, the findings must be interpreted in light of the study design. First, this was a retrospective single-center cohort, and the allocation of whole-spine versus segmental MRI was not randomized. Patients from the orthopedic trauma center underwent primary whole-spine MRI when spondylodiscitis was suspected, whereas other patients within the interdisciplinary cohort sometimes received only segmental MRI or alternative imaging. This introduces the possibility of indication bias and limits causal interpretation. In addition, the implementation of a standardized whole-spine MRI protocol during the study period (from 2015 onward) introduced a temporal practice effect, such that patients diagnosed in the later years of the study were systematically more likely to undergo whole-spine imaging. Concurrent changes in referral patterns, clinical thresholds for imaging, contemporaneous management of comorbidities, and antimicrobial practice cannot be fully accounted for in a retrospective design and may have differentially influenced both the populations imaged and the outcomes observed. Accordingly, our data demonstrate that broader MRI coverage was associated with a higher detection of multifocal disease, but they cannot establish that whole-spine MRI is superior to a targeted segmental approach in a randomized sense.

Second, the cohort was analyzed in a broad real-world manner and included all patients with imaging-confirmed infectious spondylodiscitis at first diagnosis, regardless of etiology or treatment approach. This strengthens clinical representativeness but also introduces heterogeneity with respect to etiology, baseline risk profile, diagnostic pathway, and treatment course. Additionally, a potential referral bias should be considered: as a university hospital providing maximum-level care, the institution may receive a disproportionate number of severely ill or refractory patients transferred from other hospitals, and the catchment area of a single tertiary center may limit generalizability to other settings.

Third, the analyses are descriptive: we treat multifocal involvement as a prognostic marker rather than as an exposure for causal estimation, and the design does not support claims about its independent contribution to mortality. Even within the limits of an observational, retrospective, single-center cohort, the association is clinically meaningful, because identifying multifocal disease at first imaging flags a subgroup at substantially elevated in-hospital risk.

Despite these limitations, the study has clear strengths. It includes a comparatively large cohort of 274 patients from routine clinical practice over a 10-year period, focuses on a clinically relevant diagnostic question that has been under-represented in the literature, and links imaging extent to a meaningful outcome signal. To our knowledge, this is among the larger cohorts specifically addressing the prevalence and prognostic implications of multifocal spondylodiscitis, making the present work relevant to spine surgeons involved in diagnostic strategy and treatment planning.

Future studies should aim to confirm these findings in multicenter prospective cohorts with standardized whole-spine MRI protocols, to evaluate whether early detection and targeted treatment of secondary foci translates into improved clinical outcomes, and to define more precisely which patient subgroups benefit most from extended imaging. Multivariable analyses incorporating comorbidity, pathogen type, and imaging extent in larger datasets will be needed to disentangle the independent contributions of these factors to mortality in spondylodiscitis.

## 5. Conclusions

Multifocal spondylodiscitis was detected in 18% of patients who underwent whole-spine MRI, compared with less than 2% of those examined by segmental MRI, and was associated with a mortality rate of 40%, approximately three times higher than in monosegmental disease. These findings suggest that multifocal disease is not rare, may be underdetected when imaging is restricted to the symptomatic region, and identifies a high-risk subgroup. Because not all infected spinal levels necessarily produce localizing symptoms [1,8], infection foci in other spinal regions may remain clinically silent. The results therefore support the consideration of a low threshold for whole-spine MRI in the initial diagnostic workup of suspected spondylodiscitis, particularly when disease extent may influence risk assessment and management. Confirmation in prospective multicenter cohorts is needed before broader recommendations can be made.

## Figures and Tables

**Table 1:** Patient characteristics (n = 274).

Variable	n	%
**Sex**		
Male	166	60.6
Female	108	39.4
**Age category**		
≤50 years	22	8.0
51–70 years	59	21.5
>70 years	193	70.4
**Comorbidities (n = 268)**		
Cardiovascular disease	155	56.6
Diabetes mellitus	75	27.4
Malignant disease	72	26.9
Renal disease	58	21.6
Immunosuppressive conditions	56	20.4
Liver disease or substance abuse	35	12.8
**Number of comorbidities (n = 268)**		
0	40	14.9
1	99	36.9
2	59	22.0
≥3	70	26.1
Prior spinal surgery or injection	65	23.7

Percentages calculated against total cohort (n = 274) unless otherwise noted. Bold rows indicate category headings.

**Table 2:** Disease distribution by imaging modality (n = 274).

Disease Distribution	Whole-Spine MRI (n = 139)	Segmental MRI (n = 116)	Other Imaging (n = 19)	Total (n = 274)
Monosegmental	84 (60.4%)	98 (84.5%)	16 (84.2%)	198 (72.3%)
Multisegmental unifocal	30 (21.6%)	16 (13.8%)	0 (0.0%)	46 (16.8%)
Multifocal	25 (18.0%)	2 (1.7%)	3 (15.8%)	30 (10.9%)

Values are n (column %). Other imaging includes CT and bone scintigraphy.

**Table 3:** In-hospital mortality by disease distribution, comorbidity burden, pathogen, and sex.

Subgroup	n	Deaths	Mortality (%)	*p*-Value	Cramér’s V
**Disease distribution**				**0.002**	**0.22**
Monosegmental	197	27	13.7		
Multisegmental unifocal	45	7	15.6		
Multifocal	30	12	40.0		
**Number of comorbidities**				**0.008**	**0.20**
0	40	3	7.5		
1	99	10	10.1		
2	59	12	20.3		
≥3	70	19	27.1		
**Pathogen**				**0.010**	**0.20**
*S. aureus*	65	17	26.2		
No pathogen identified	109	12	11.0		
**Sex**				**0.771**	**—**
Male	165	29	17.6		
Female	107	17	15.9		

*p*-values from chi-square test. Mortality denominator: n = 272 (disease distribution, sex), n = 268 (comorbidity), n = 174 (pathogen). Bold rows indicate category headings.

## Data Availability

Data are available from the corresponding author upon reasonable request.
